# Interaction of some phytochemical compounds with Er2O3 nanoparticle: First principle study

**DOI:** 10.1007/s00894-025-06361-4

**Published:** 2025-04-03

**Authors:** Mahmood Akbari

**Affiliations:** UNESCO‑UNISA‑ITL/NRF Africa Chair in Nanoscience & Nanotechnology (U2ACN2), College of Graduate Studies, University of South Africa (UNISA), Pretoria, South Africa

**Keywords:** Er2O3 nanoparticles, DFT calculations, Phytochemical compounds, MD simulation, Solubility

## Abstract

**Context:**

The interaction between phytochemicals and nanoparticles plays a crucial role in nanotechnology and biomedical applications. This study investigates the binding behavior and stability of six phytochemicals—Catechin, Limonene, Sabinene, Sinapic Acid, Vanillic Acid, and Luteolin 7-O-ß-glucuronide—with Er₂O₃ nanoparticles using Density Functional Theory (DFT) and Molecular Dynamics (MD) simulations. The findings indicate that Luteolin, Catechin, and Sinapic Acid exhibit the strongest binding affinities and highest structural stability with Er_2_O_3_, attributed to their balanced hydrophilicity-lipophilicity and favorable electronic properties. These insights contribute to the design and functionalization of phytochemical-based nanomaterials, with potential applications in drug delivery, bioimaging, and photodynamic therapy.

**Methods:**

DFT calculations were conducted using Gaussian 09 at the B3LYP/6–311 +  + G(d,p) level to determine HOMO–LUMO energy gaps, dipole moments, and polarizability of the phytochemicals. MD simulations, performed using GROMACS 2019 with the CHARMM36 force field and TIP3P water model, analyzed the dynamics of phytochemical adsorption on a 5 nm Er_2_O_3_ nanoparticle over 50 ns. Key parameters such as interaction energies, root mean square deviations (RMSD), radial distribution functions (RDF), and water solubility (logS) were evaluated using ALOPGPS 2.1 software.

## Introduction

Phytochemicals, a diverse class of bioactive compounds produced by plants, play a crucial role in color, flavor, and disease resistance while offering numerous therapeutic benefits [1, 2]. Found in fruits, vegetables, grains, and beans, these compounds have been widely studied for their antioxidant [3, 4], anti-inflammatory [5], antimicrobial [6], and immune-boosting properties [7]. Their bioactivities make them valuable in pharmacology, nutrition, and cosmetics, as they contribute to chronic disease prevention [8], including cancer [9], and cardiovascular diseases [10], and neurodegenerative disorders [11]. Additionally, phytochemicals have shown cardioprotective advantages [12] and hormonal regulatory effects [3], serving as promising natural alternatives to synthetic medications [13], further expanding their use in therapeutic applications [14], dietary supplements [15], functional foods [16, 17] and cosmetic formulations [18, 19].

Among phytochemicals, phenolic compounds [20] and terpenes [21] are particularly significant due to their biological and chemical versatility. Phenolic compounds, including Catechin, Sinapic acid, Vanillic acid, and Luteolin 7-O-ß-glucuronide exhibit strong antioxidant activity, making them potential candidates for cancer prevention and cardiovascular health support [22, 23]. On the other hand, terpenes such as Limonene and Sabinene, known for their distinct aromatic properties, display anti-inflammatory, antimicrobial, and antiviral activities [24–27]. These bioactive compounds not only contribute to plant defense mechanisms but also hold promise in biomedical and pharmaceutical applications, particularly in nanotechnology-driven drug delivery and therapeutic interventions [28].

Erbium oxide (Er_2_O_3_) nanoparticles have gained attention due to their exeptional optical [29] and electronic properties [30], finding applications in telecommunications [31] and biomedicine [32]. Functionalizing Er₂O₃ nanoparticles with phytochemicals has been shown to improve solubility, biocompatibility, and therapeutic efficacy [33–35]. These functionalized nanoparticles enable targeted drug delivery, allowing for precise interaction with specific tissues or cells, such as cancer cells, while minimizing side effects. Additionally, Er₂O₃ nanoparticles have shown potential in bioimaging, where their optical properties enhance imaging sensitivity for early disease detection [36, 37]. In photodynamic therapy (PDT), these nanoparticles act as photosensitizers, generating reactive oxygen species (ROS) upon light exposure, selectively destroying cancer cells [38–40]. The synergistic combination of Er₂O₃ nanoparticles with phenolic compounds and terpenes not only enhances nanoparticle stability and bioavailability but also facilitates controlled drug release, improving overall therapeutic outcomes.

To comprehensively investigate these interactions, this study employs a dual computational approach using Density Functional Theory (DFT) and Molecular Dynamics (MD) simulations. DFT calculations provide insights into the electronic properties, chemical reactivity, and active binding sites of the studied phytochemicals, while MD simulations evaluate the dynamic stability and adsorption behavior of these molecules on Er₂O₃ nanoparticles in a solvated environment [41–44]. By integrating these computational techniques, we aim to uncover the fundamental mechanisms governing phytochemical-nanoparticle interactions, offering valuable insights into their potential nanomedicine applications.

### Computational method

To comprehensively investigate the interaction behavior of the studied phytochemicals with Er₂O₃ nanoparticles, a two-step computational approach was employed, combining Density Functional Theory (DFT) calculations and Molecular Dynamics (MD) simulations. First, DFT was utilized to analyze the electronic structure, molecular reactivity, and key interaction sites of the phytochemicals, providing a fundamental understanding of their properties. Then, MD simulations were performed to explore the dynamic adsorption behavior, stability, and solvation effects of these compounds on the Er₂O₃ nanoparticle in an aqueous environment. This integrated approach allows for both quantum mechanical insight into reactivity and large-scale simulation of realistic molecular interactions.

### Density functional theory (DFT) calculations

DFT calculations were conducted using Gaussian 09 software [45] to determine the electronic properties, chemical reactivity, and potential active sites of the six studied phytochemicals: Catechin, Limonene, Sabinene, Sinapic Acid, Vanillic Acid, and Luteolin 7-O-β-glucuronide. Geometry optimization was performed at the B3LYP/6–311 +  + G(d,p) level, which provides a balance between computational efficiency and accuracy in modeling molecular electronic properties [46]. Although B3LYP is known to overestimate HOMO–LUMO gaps, it remains widely used for evaluating molecular reactivity and charge transfer behavior, offering reliable insights into molecular interactions with nanoparticles [47].

To ensure structural stability, each optimized molecule was subjected to vibrational frequency analysis, confirming the absence of imaginary frequencies, which indicates that the structures correspond to true minima on the potential energy surface (PES). The HOMO–LUMO energy gap (*E*_*gap*_ = *E*_*LUMO*_*—E*_*HOMO*_) was calculated for each molecule, serving as an indicator of chemical reactivity, where a lower gap suggests a higher tendency for charge transfer and interaction [48].

In addition to frontier molecular orbitals, several quantum molecular descriptors were derived, including chemical potential (*μ* = *(E*_*LUMO*_ + *E*_*HOMO*_*) /2*), chemical hardness (*η* = *(E*_*LUMO*_*—E*_*HOMO*_*) / 2*), electronegativity (*χ* = *-μ*), and electrophilicity index (*ω* = *χ*^*2*^* / 2η*) [48, 49]. These descriptors provide valuable insight into the compounds’ stability, reactivity, and interaction potential with the Er₂O₃ nanoparticle [48].

To further identify active binding sites, molecular electrostatic potential (MEP) maps were generated. These maps visualize electron density distribution, where electron-rich (nucleophilic) regions are favorable for interactions with electrophilic surfaces such as Er_2_O_3_, while electron-deficient (electrophilic) areas influence binding orientations [50]. The DFT results laid the foundation for understanding how each phytochemical interacts at the electronic level, guiding the subsequent molecular dynamics simulations.

### Molecular DYNAMICS (MD) simulations

**Fig. 1:**
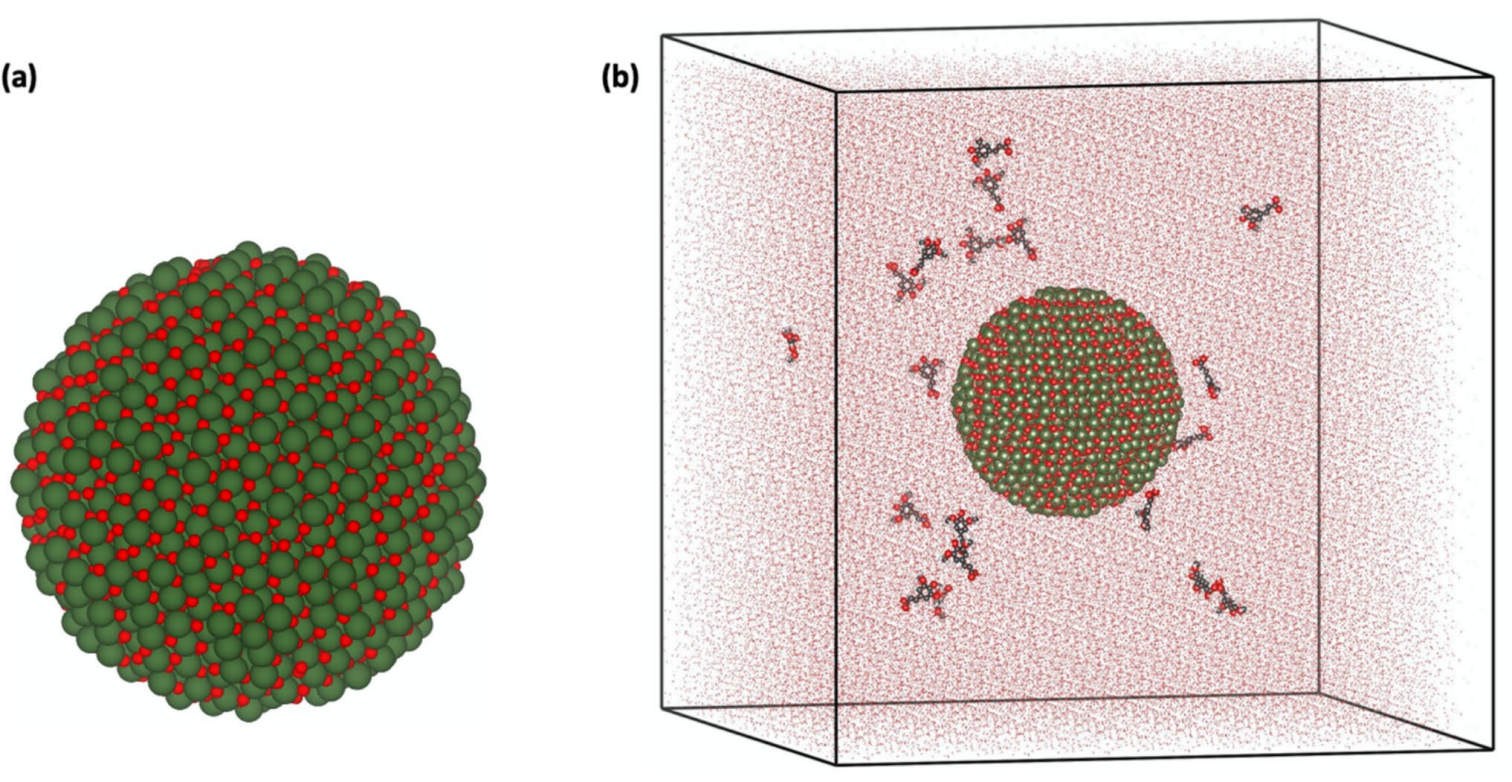
Schematic view of (**a**) an Er_2_O_3_ spherical nanoparticle. (**b**) an Er_2_O_3_ nanoparticle surrounded with 20 molecules of Catechin and water molecules in a simulation box

Building upon the DFT findings, MD simulations were performed to explore the dynamic interaction behavior, structural stability, and adsorption properties of the phytochemicals on a spherical Er_2_O_3_ nanoparticle in a solvated environment. The Er_2_O_3_ nanoparticle (5 nm diameter, 4463 atoms) was generated using Atomsk software [51] and positioned at the center of a cubic simulation box (8 × 8 × 8 nm^3^), as shown in Fig. 1(a). The nanoparticle’s Lennard–Jones (LJ) parameters were obtained from previously published studies [52, 53], ensuring an accurate representation of its interactions.

To model adsorption, 20 molecules of each phytochemical were introduced into the simulation box with random initial placements, maintaining a minimum 1 nm distance from the box edges to avoid boundary interference (Fig. 1(b)). The system was solvated using TIP3P water molecules to simulate realistic hydration conditions [54].

MD simulations were conducted using GROMACS 2019 [55] with the CHARMM36 force field [56], which has been extensively validated for biomolecular interactions. The CHARMM General Force Field (CGenFF) was used to parameterize the phytochemicals [57], while Lennard–Jones (LJ) parameters for Er₂O₃ were incorporated from previous studies [52, 53] to model van der Waals interactions between the nanoparticle and phytochemicals.

To remove unfavorable steric clashes, energy minimization was carried out using the steepest descent algorithm [58]. The system was equilibrated in two phases: first, in the NVT ensemble (constant Number, Volume, and Temperature) at 300 K for 100 ps using the V-rescale thermostat, followed by the NPT ensemble (constant Number, Pressure, and Temperature) at 1 atm for 200 ps using the Berendsen barostat. After equilibration, 50 ns production MD simulations were performed under constant pressure (1 atm) and temperature (300 K) with a 2 fs time step. Periodic boundary conditions (PBCs) were applied to eliminate edge effects, and long-range electrostatic interactions were computed using the Particle Mesh Ewald (PME) method [59]. The LINCS algorithm was employed to constrain hydrogen bond lengths [60].

Post-simulation analyses were conducted using GROMACS utilities to evaluate the binding stability and adsorption behavior of the phytochemicals. The root mean square deviation (RMSD) provided insights into the stability of each molecule upon adsorption, while the radial distribution function (RDF) was used to determine the preferential binding distances between the phytochemicals and the nanoparticle. Interaction energies, including van der Waals and electrostatic contributions, were extracted to quantify the adsorption strength. The visualization was conducted with VMD 1.9.3 [61]. Additionally, the water solubility (logS) of each compound was calculated using ALOGPS 2.1 software [62] to assess the role of hydrophilicity-lipophilicity balance in molecular interactions.

The combination of DFT and MD methodologies provided a comprehensive perspective on the phytochemical-Er₂O₃ interactions. While DFT offered a quantum mechanical understanding of electronic properties, charge transfer potential, and reactive binding sites, MD simulations enabled the real-time evaluation of adsorption stability, molecular flexibility, and solvation effects. Given the large size of the Er₂O₃ nanoparticle (5 nm, 4463 atoms), a full DFT treatment was computationally prohibitive, making MD simulations a more practical and scalable approach to studying realistic interaction dynamics. This hybrid computational strategy ensures that both fundamental electronic properties and large-scale dynamic behavior are accurately captured, providing valuable insights into the functionalization of Er_2_O_3_ nanoparticles for biomedical applications.

## Results and discussions

**Fig. 2:**
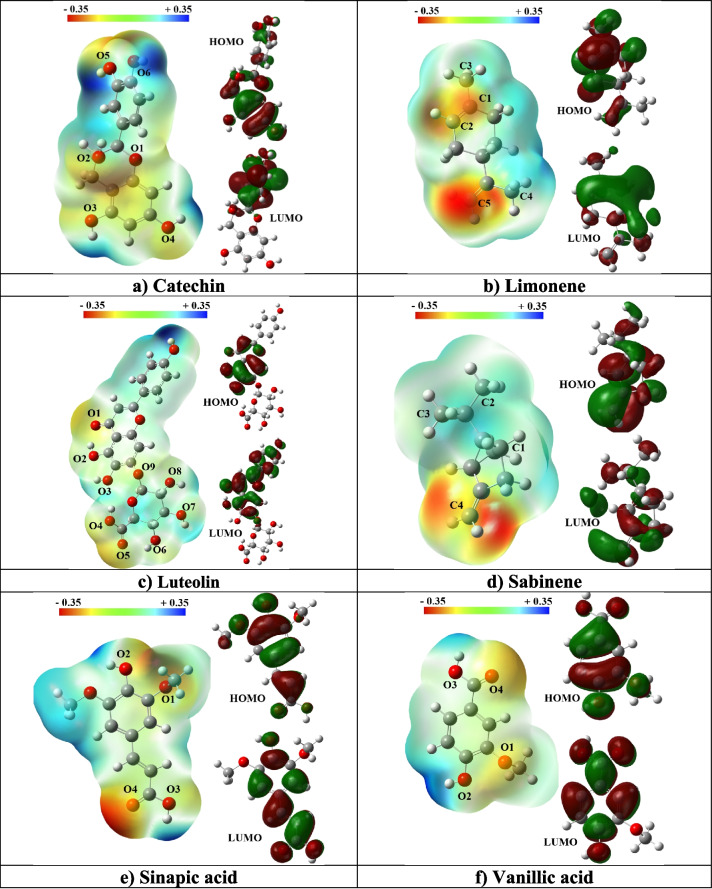
HOMO and LUMO orbitals and molecular electrostatic potential (MEP) map for the studied compounds

The optimized molecular structures of the six selected phytochemicals—Catechin, Limonene, Luteolin, Sabinene, Sinapic Acid, and Vanillic Acid—as illustrated in Fig. 2, exhibit structural stability, as confirmed by the absence of imaginary frequencies in their vibrational frequency analyses. This validation confirms that each structure represents a true energy minimum on the potential energy surface [63], making them suitable for further quantum and classical interaction studies with Er_2_O_3_ nanoparticles.

The DFT-derived properties, including total energy, dipole moment, polarizability, HOMO–LUMO energy gap, and molecular electrostatic potential (MEP), provide a foundational understanding of the electronic behavior and reactivity of these compounds. These descriptors are particularly important in predicting the nature and strength of interaction between the phytochemicals and the Er₂O₃ nanoparticle, which is essential for nanomaterial design and biomedical applications.

As shown in Table 1, Luteolin exhibits the lowest total energy (−1714.313 Hartree) among the studied compounds, suggesting it is the most thermodynamically stable [64]. In contrast, Limonene and Sabinene exhibit significantly higher total energies (−390.781 and −390.763 Hartree, respectively), indicating comparatively lower molecular stability. This trend correlates with the molecular complexity and functional group density. With its highly conjugated system and multiple hydroxyl groups, Luteolin benefits from enhanced electronic delocalization and hydrogen bonding, both of which contribute to its structural robustness. Similar structure-stability relationships have been discussed in prior computational studies on flavonoids and phenolic acids [65].

**Table 1:** Total energy (Hartree), Dipole moment (Debye), and polarizability (a.u) for the optimized structures at the B3LYP/6–311 +  + g(d,p) level of theory

Compounds	Total energy	Total enthalpy	Total Gibbs free	Dipole moment	Polarizability
Catechin	−1031.697178	−1031.408441	−1031.473700	1.507326	290.795333
Limonene	−390.780964	−390.536065	−390.584087	1.266214	172.790667
Luteolin	−1714.313320	−1713.907472	−1714.000755	10.134597	467.105333
Sabinene	−390.762993	−390.518600	−390.564965	1.473607	170.285333
Sinapic acid	−802.748194	−802.515378	−802.577758	4.403937	253.298667
Vanillic acid	−610.766798	−610.604237	−610.653887	5.936003	163.230667

Polarizability further reflects a molecule’s capacity to interact with an external electric field [66], such as that presented by the polar surface of Er₂O₃. Luteolin again exhibits the highest polarizability (467.11 a.u.), suggesting greater adaptability and stronger van der Waals interactions with the nanoparticle. In contrast, Vanillic Acid, with the lowest polarizability (163.23 a.u.), is expected to have weaker surface interactions. These findings align with previous reports where higher polarizability enhanced adsorption onto oxide surfaces [67].

Dipole moment, a measure of molecular polarity, is also indicative of interaction potential, particularly with polar inorganic surfaces like Er₂O₃. Luteolin’s dipole moment (10.13 Debye) suggests a strong electrostatic interaction potential, while Limonene (1.27 Debye) is far less polar and thus less likely to stably adsorb on the nanoparticle.

The frontier molecular orbital analysis (Table 2) offers deeper insight into the compounds’ chemical reactivity and charge transfer capabilities. Luteolin (*E*_*gap*_ = 3.95 eV) and Catechin (5.56 eV) show smaller HOMO–LUMO energy gaps, indicating a higher potential for electron exchange and surface adsorption. In contrast, Limonene and Sabinene, with larger gaps (6.23 and 6.27 eV), are less chemically reactive, which is expected to limit their interaction strength with Er_2_O_3_. These values are consistent with previous studies showing that flavonoids with low E_*gap*_ values interact more readily with metal oxide surfaces [68].

**Table 2:** The HOMO and LUMO energy, energy gap (eV), chemical potential ($\mu$), chemical hardness ($\eta$), chemical softness (s), electronegativity ($\chi$), and electrophilicity ($\omega$), (in eV) of investigated phytocompounds in water solvent

Compounds	${E}_{HOMO}$	${E}_{LUMO}$	${E}_{gap}$	$\mu$	$\eta$	$s$	$\chi$	$\omega$
Catechin	−6.09	−0.52	5.56	−3.30	2.78	0.36	3.30	1.96
Limonene	−6.32	−0.09	6.23	−3.21	3.11	0.32	3.21	1.65
Luteolin	−6.19	−2.24	3.95	−4.21	1.97	0.51	4.21	4.50
Sabinene	−6.31	−0.04	6.27	−3.17	3.13	0.32	3.17	1.61
Sinapic acid	−6.05	−2.17	3.87	−4.11	1.94	0.52	4.11	4.36
Vanillic acid	−6.69	−1.65	5.03	−4.17	2.52	0.40	4.17	3.45

Additionally, chemical hardness (η), which is inversely proportional to reactivity, supports these trends: Luteolin (1.97 eV) and Sinapic Acid (1.94 eV) show the lowest hardness, making them the most chemically soft and reactive candidates. This softness implies greater susceptibility to polarization and stronger surface binding, as also observed in DFT-based nanomaterial interaction studies [69].

Other reactivity descriptors such as chemical potential (μ), electronegativity (χ), and electrophilicity index (ω) provide further insight. Notably, Luteolin (ω = 4.50 eV) and Sinapic Acid (ω = 4.36 eV) exhibit high electrophilicity, indicating a strong ability to accept electrons from donor species, such as the oxide surface. These properties point to their favorable interaction characteristics, aligning well with the theoretical framework used to predict efficient surface conjugation in nanomedicine [48, 49].

The Molecular Electrostatic Potential (MEP) maps presented in Fig. 2 provide valuable insights into the charge distribution and potential interaction sites of the studied phytochemicals. These maps visually highlight electron-rich (nucleophilic) and electron-poor (electrophilic) regions, thereby identifying likely sites for interaction with electrophilic species such as Er_2_O_3_ nanoparticles.

For Catechin, the MEP reveals intense red zones around the hydroxyl oxygen atoms (O1–O5), suggesting strong electron density and high nucleophilicity. These regions are highly favorable for electrostatic and hydrogen bonding interactions with the positively polarized regions on the Er_2_O_3_ surface. Similar behavior is seen in Luteolin, which displays pronounced red areas around hydroxyl and carbonyl groups (O1, O5, O6, and O7), indicating its potential for hydrogen bonding and charge transfer interactions—critical for surface adsorption and stabilization in nanoconjugates [70].

In contrast, Limonene shows red regions near its C4–C5 double bond, indicating the location of its π-electrons. However, its low overall polarity and absence of polar functional groups limit its capacity for strong interactions with polar or ionic nanoparticle surfaces, such as Er_2_O_3_. Sabinene exhibits a similar distribution, with localized reactivity around its double bond (C4), but lacks the hydroxyl or carboxyl functionalities needed for more effective interactions. This observation aligns with previous findings indicating that nonpolar terpenes exhibit reduced binding affinity to oxide surfaces due to limited hydrogen bonding capacity [71].

Sinapic Acid and Vanillic Acid, on the other hand, show strong red regions around their carboxylic and hydroxyl groups (O4 for Sinapic Acid; O3 and O4 for Vanillic Acid), which are well-known active sites for interaction with metal oxide surfaces. These electron-rich regions suggest favorable electrostatic and hydrogen bond-driven interactions with Er_2_O_3_ nanoparticles, in agreement with prior reports on phenolic acid interactions with metal oxides [72].

Analysis of the HOMO and LUMO distributions further supports these observations. In Catechin, Luteolin, Sinapic Acid, and Vanillic Acid, the HOMO is delocalized over aromatic rings and hydroxyl groups, indicating a strong tendency to donate electrons, which facilitates charge transfer to electrophilic nanoparticle surfaces. The LUMO orbitals of these molecules are also distributed over aromatic and polar regions, enabling charge delocalization upon interaction. This electronic structure promotes both donor–acceptor interactions and π–π stacking, strengthening binding potential with the Er_2_O_3_ surface.

In contrast, the HOMO and LUMO in Limonene and Sabinene are largely restricted to localized π-bonds, particularly around the double bonds. Their limited orbital overlap with polar or charged species reduces their versatility and reactivity in forming strong and stable complexes with Er_2_O_3_.

Taken together, the results confirm that Luteolin, Catechin, Sinapic Acid, and Vanillic Acid possess the most favorable electronic and structural characteristics for interacting with Er_2_O_3_ nanoparticles. These include high electron density at reactive sites, low HOMO–LUMO energy gaps, high polarizability, and strong dipole moments, all of which contribute to enhanced surface affinity and potential stability in nanomaterial formulations. Among these, Luteolin stands out as the most promising candidate, owing to its exceptional polarizability (467.11 a.u.), high dipole moment (10.13 D), and lowest energy gap (3.95 eV). These properties suggest it is not only highly reactive but also well-suited for charge transfer and strong surface adsorption.

In contrast, Limonene and Sabinene, despite exhibiting some localized reactivity through their double bonds, are overall less favorable due to their low polarity, minimal functional group diversity, and restricted orbital distributions. These findings are consistent with prior studies that emphasize the importance of hydroxylation and aromaticity in enhancing nanoparticle-plant compound interactions [73].

The logS values presented in Table 3, calculated using ALOGPS 2.1 software [62], offer key insights into the aqueous solubility and hydrophilic-lipophilic balance of the studied phytochemicals. Defined as the base-10 logarithm of molar solubility in water, logS values are instrumental in assessing a compound’s dispersion potential in biological environments and its affinity for polar or nonpolar surfaces. Compounds with logS values between −1 and −5 are considered to exhibit a balanced hydrophilic-lipophilic character, allowing for adequate solubility in water while retaining the ability to interact with hydrophobic or polar surfaces, such as metal oxide nanoparticles [62, 74].

**Table 3:** LogS values calculated for six active compounds

Compounds	Catechin	Limonene	Luteolin	Sabinene	Sinapic acid	Vanillic acid
LogS	−2.65	−3.11	−2.52	−3.33	−2.55	−1.47

Among the studied compounds, Vanillic Acid shows the highest water solubility (logS = –1.47), attributed to its hydroxyl and carboxylic functional groups, as evident in Fig. 2. While this high hydrophilicity supports strong interaction with water, it reduces the molecule’s tendency to adsorb onto Er₂O₃ nanoparticles, as highly soluble molecules often remain preferentially solvated in the aqueous phase. This interpretation is consistent with Vanillic Acid’s moderate dipole moment (5.94 Debye) and low polarizability (163.23 a.u.) from Table 1, which together suggest a strong affinity for water and limited adaptability to nanoparticle surfaces.

In contrast, Catechin (logS = –2.65) and Luteolin (logS = –2.52) demonstrate intermediate solubility, striking an optimal balance between hydrophilicity and lipophilicity. This balance favors adsorption onto polar surfaces such as Er₂O₃ while maintaining sufficient solubility for biological compatibility. Both compounds exhibit high polarizability (290.80 a.u. for Catechin and 467.11 a.u. for Luteolin) and significant electron density on hydroxyl and carbonyl groups (Fig. 2), making them highly responsive to external electric fields and polar surface charges. These features enhance their adsorption potential. Moreover, Luteolin’s smaller HOMO–LUMO gap (3.95 eV) (Table 2) indicates a higher charge transfer capacity, further supporting its strong interaction potential with Er₂O₃.

Sinapic Acid (logS = –2.55) also demonstrates a well-balanced solubility profile. Its carboxylic and hydroxyl groups, visualized in Fig. 2, promote interactions with polar surfaces, while its dipole moment (4.40 Debye) and polarizability (253.30 a.u.) suggest adaptability to both aqueous and nanoparticle environments. Its low HOMO–LUMO gap (3.87 eV) further enhances its reactivity, positioning it alongside Luteolin and Catechin as a strong candidate for Er_2_O_3_ interaction.

At the opposite end of the solubility spectrum, Limonene (logS = –3.11) and Sabinene (logS = –3.33) are the least hydrophilic and most lipophilic compounds in this study. Their non-polar structures and limited functional groups (Fig. 2) result in reduced water solubility but enhanced affinity for nonpolar environments. While this lipophilic character may promote adsorption onto less polar surfaces, Er₂O₃’s polar nature requires a degree of molecular polarity and functional diversity for strong and stable binding. Their low dipole moments (1.27 Debye for Limonene and 1.47 Debye for Sabinene) and large HOMO–LUMO gaps (6.23 and 6.27 eV, respectively) indicate limited reactivity and charge transfer ability, reducing their effectiveness in nanoparticle binding despite favorable lipophilicity.

In summary, this analysis underscores that intermediate logS values, as observed for Luteolin, Catechin, and Sinapic Acid, are optimal for effective Er₂O₃ interaction, offering both sufficient aqueous solubility for dispersion and strong surface affinity for adsorption. In contrast, highly hydrophilic compounds like Vanillic Acid may remain solvated and interact less strongly with Er₂O₃, while highly lipophilic but weakly reactive compounds like Limonene and Sabinene lack the electronic features required for effective binding. Taken together, these findings reinforce that Luteolin, Catechin, and Sinapic Acid are the most promising candidates for nanoparticle surface functionalization, particularly in aqueous nanomedicine formulations where balanced solubility and high reactivity are critical.

**Fig. 3:**
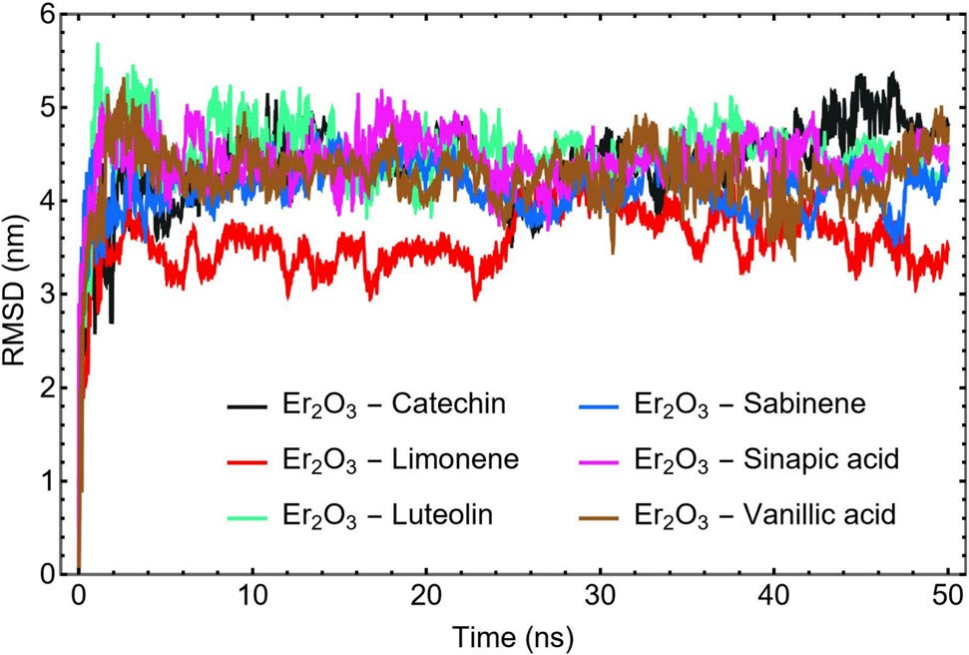
RMSD plots of the phytochemical compounds with Er_2_O_3_ nanoparticles. MD trajectories beyond the 10 ns were selected for further analysis

The Root Mean Square Deviation (RMSD) profiles, depicted in Fig. 3, provide key insights into the structural stability and dynamic behavior of the studied phytochemicals during their 50 ns molecular dynamics (MD) simulations in the presence of Er_2_O_3_ nanoparticles. The initial sharp rise in RMSD corresponds to the equilibration phase, where the molecules undergo conformational adjustments as they respond to the surrounding solvent and interact with the nanoparticle surface. Following this early phase, most compounds reach a plateau, indicating the attainment of dynamic equilibrium.

Among the studied molecules, Catechin, Luteolin, and Sinapic Acid exhibit low and stable RMSD values throughout the production run, suggesting minimal conformational fluctuations and strong, consistent interactions with the Er₂O₃ surface. This dynamic stability reflects their favorable physicochemical characteristics, including moderate water solubility (logS values around –2.5), high dipole moments, and high polarizability (Table 1), as well as low HOMO–LUMO gaps, which collectively enhance their binding affinity and adaptability to polar surfaces. These observations are in line with earlier findings in similar MD studies, where flavonoids with hydroxyl-rich structures and balanced polarity maintained stable adsorption onto oxide nanoparticle surfaces [75].

To ensure that the analysis was performed on fully equilibrated systems, only the MD trajectories after the initial 10 ns were used for subsequent interaction energy and structural analyses. This approach enhances the reliability and consistency of the simulation data.

In contrast, Limonene and Sabinene demonstrate higher RMSD values and greater fluctuations, indicating reduced stability and weaker interactions with the nanoparticle surface. These patterns are consistent with their nonpolar, hydrophobic structures, limited functional group availability, and low dipole moments, which hinder strong electrostatic or hydrogen-bonding interactions with the polar Er₂O₃ surface. Their behavior suggests that, although they may weakly adsorb onto the nanoparticle via van der Waals interactions, the lack of electronic complementarity limits their binding strength and stability.

Vanillic Acid exhibits intermediate RMSD behavior, reflecting moderate binding potential and conformational flexibility. While it possesses polar functional groups conducive to surface interaction, its high aqueous solubility and relatively low polarizability likely reduce its persistence on the nanoparticle surface.

**Fig. 4:**
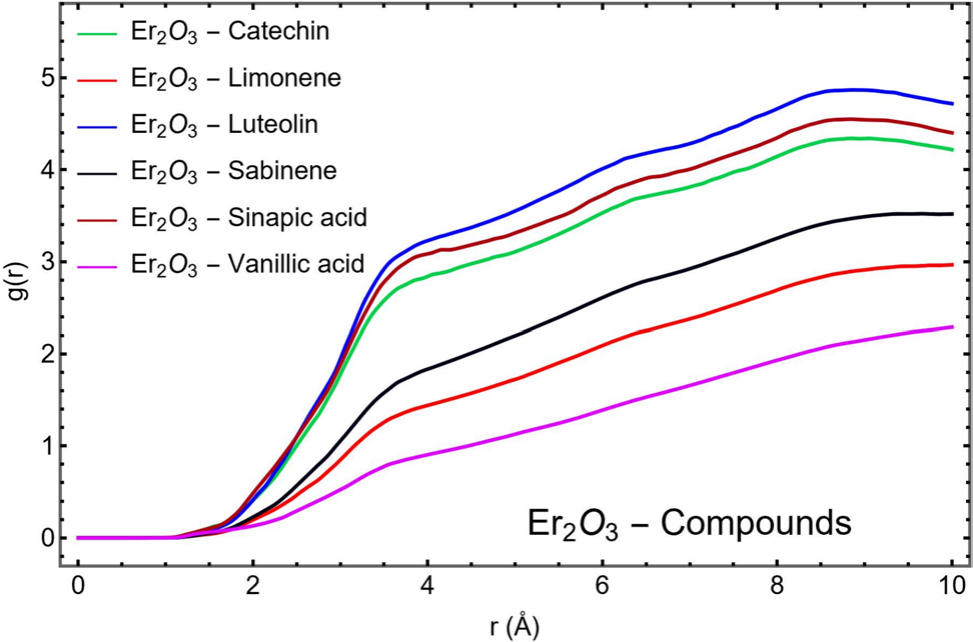
RDF plot of six phytochemical compounds with respect to the Er_2_O_3_ nanoparticle

The Radial Distribution Function (RDF) profiles, presented in Fig. 4, offer critical insights into the spatial organization and interaction strength of the studied phytochemicals in relation to the Er_2_O_3_ nanoparticle surface. RDF, denoted as g(r), describes the probability of finding an atom of a given compound at a distance r from a reference point on the Er_2_O_3_ surface compared to an ideal, randomly distributed system. Peaks in the RDF indicate preferred distances of interaction, with the height and sharpness of the peak reflecting the strength and specificity of the interaction.

The first coordination peak in each RDF curve signifies the most probable distance of closest interaction between the compound and the nanoparticle surface. Among all studied molecules, Luteolin exhibits the highest and sharpest first peak, indicating a strong, well-defined, and specific interaction with the Er_2_O_3_ surface. This behavior is attributed to Luteolin's high dipole moment, large polarizability, and the presence of multiple polar functional groups, particularly hydroxyl and carbonyl moieties, which are capable of forming hydrogen bonds and electrostatic interactions with surface atoms.

Catechin and Sinapic Acid also exhibit pronounced and sharp RDF peaks, though slightly lower than Luteolin, reflecting significant and stable interactions with Er_2_O_3_. These findings are consistent with their balanced hydrophilic-lipophilic nature, moderate water solubility, and high electronic reactivity, as observed in their HOMO–LUMO gaps and quantum descriptors (Tables 1 and 2). The presence of aromatic systems and polar substituents in these molecules contributes to strong adsorption behavior, in line with prior studies demonstrating the role of hydroxyl-rich phenolics in surface functionalization of metal oxides [76].

In contrast, Sabinene and Limonene show broader and lower RDF peaks, indicating weaker, less specific interactions with the nanoparticle surface. These compounds, being nonpolar and hydrophobic, lack the functional groups necessary for stable electrostatic or hydrogen-bonding interactions. Their greater conformational flexibility and lack of defined orientation during binding contribute to the observed distribution over a wider range of distances, a hallmark of transient or non-specific interactions.

Vanillic Acid presents the lowest and broadest RDF peak, suggesting the weakest surface interaction among the six compounds. Although it possesses polar functional groups, its high water solubility and relatively low polarizability likely favor solvent interactions over nanoparticle binding, reducing its effective surface contact with Er₂O₃. This is in agreement with its moderate dipole moment and lowest interaction energy observed in earlier MD and DFT analyses.

Beyond the first coordination shell, all compounds show g(r) values approaching unity, indicating that at larger distances, the molecular distribution becomes uniform, as expected in a well-equilibrated, solvated system. This trend confirms that interactions are localized near the nanoparticle surface, with limited long-range structuring in the bulk phase.

The order of interaction strength, inferred from the height and sharpness of the first RDF peak, can be ranked as follows:

$$Luteolin\hspace{0.17em}>\hspace{0.17em}Catechin\hspace{0.17em}>\hspace{0.17em}Sinapic\, Acid\hspace{0.17em}>\hspace{0.17em}Sabinene\hspace{0.17em}>\hspace{0.17em}Limonene\hspace{0.17em}>\hspace{0.17em}Vanillic\, Acid$$

This ranking aligns closely with trends in dipole moments, polarizability, HOMO–LUMO energy gaps, and logS values, reinforcing the conclusion that Luteolin, Catechin, and Sinapic Acid exhibit the most favorable properties for strong and stable interactions with Er_2_O_3_ nanoparticles. Their consistent performance across quantum, structural, and dynamic descriptors supports their suitability for surface functionalization in applications such as targeted drug delivery, biosensing, or photodynamic therapy.

Conversely, while Limonene and Sabinene show some surface affinity due to their lipophilic nature, their lack of polar functionality and low electronic reactivity limits their versatility and binding strength. Vanillic Acid, although hydrophilic and polar, demonstrates the lowest nanoparticle interaction due to its strong solvation preference and limited adaptability to the Er_2_O_3_ surface environment.

The interaction energies computed from the molecular dynamics (MD) simulations, as presented in Table 4, offer quantitative insight into the binding affinity of each phytochemical for the Er_2_O_3_ nanoparticle surface, as well as their relative preference for aqueous versus surface environments. These interaction energies include both van der Waals and electrostatic components, providing a holistic view of the non-covalent forces governing molecular adsorption and solvation.

**Table 4:** Interaction energies extracted from MD simulations

Interaction Energies (Van der Waals + Electrostatic potential energies)		(kJ/mol)
Er_2_O_3_ NP – Catechin	Er_2_O_3_ NP—Catechin	−1026.10 ± 11
	Catechin—Water	−882.31 ± 21
Er_2_O_3_ NP – Limonene	Er_2_O_3_ NP—Limonene	−953.33 ± 13
	Limonene—Water	−1753.46 ± 24
Er_2_O_3_ NP – Luteolin	Er_2_O_3_ NP—Luteolin	−1089.77 ± 17
	Luteolin—Water	−674.45 ± 25
Er_2_O_3_ NP – Sabinene	Er_2_O_3_ NP—Sabinene	−947.36 ± 3
	Sabinene—Water	−1252.45 ± 22
Er_2_O_3_ NP – Sinapic acid	Er_2_O_3_ NP—Sinapic acid	−1073.1 ± 21
	Sinapic acid—Water	−743.38 ± 25
Er_2_O_3_ NP – Vanillic acid	Er_2_O_3_ NP—Vanillic acid	−844.42 ± 13
	Vanillic acid—Water	−2448.09 ± 35

Among all compounds, Luteolin shows the strongest interaction with Er₂O₃, with a binding energy of –1089.77 ± 17 kJ/mol. This high affinity can be attributed to its multiple hydroxyl and carbonyl functional groups, high polarizability (467.11 a.u.), and moderate water solubility (logS = –2.52). These properties allow Luteolin to effectively balance hydrophilic and lipophilic interactions, facilitating robust and specific adsorption onto the polar Er₂O₃ surface. Its interaction with water, however, is significantly weaker (–674.45 ± 25 kJ/mol), further emphasizing its preferential binding to the nanoparticle.

Catechin follows closely, with an Er_2_O_3_ interaction energy of –1026.10 ± 11 kJ/mol. Its hydroxyl-rich aromatic structure promotes strong hydrogen bonding and electrostatic interactions with the nanoparticle, consistent with its stable RMSD and sharp RDF peak (Fig. 3 and 4). Catechin also shows a lower interaction with water (–882.31 ± 21 kJ/mol) compared to its binding with Er₂O₃, reinforcing its surface selectivity and compatibility with polar nanomaterials.

Sinapic Acid also exhibits a high binding energy to Er₂O₃ (–1073.10 ± 21 kJ/mol), indicating a favorable interaction driven by its carboxylic acid and methoxy substituents, which act as strong nucleophilic sites (Fig. 2). While its interaction is slightly lower than that of Luteolin and Catechin, its water interaction energy (–743.38 ± 25 kJ/mol) suggests a similar hydrophilic-lipophilic balance, making it a strong contender for nanoparticle functionalization.

In contrast, Limonene and Sabinene exhibit weaker interactions with Er₂O₃ (–953.33 ± 13 kJ/mol and –947.36 ± 3 kJ/mol, respectively), reflective of their nonpolar, hydrocarbon-based structures and lack of polar functional groups. Notably, Limonene demonstrates a stronger interaction with water (–1753.46 ± 24 kJ/mol) than with Er₂O₃, indicating a preference for solvation over adsorption, which correlates with its moderate logS (–3.11) and low dipole moment (1.27 Debye). Similarly, Sabinene, with an interaction energy of –1252.45 ± 22 kJ/mol with water, also favors the aqueous environment, suggesting limited nanoparticle binding potential despite its slight lipophilic character.

Vanillic Acid shows the weakest interaction with Er_2_O_3_ (–844.42 ± 13 kJ/mol) among all compounds studied. This low interaction energy is consistent with its high water affinity, as evidenced by its very strong water interaction energy of –2448.09 ± 35 kJ/mol, the highest among the group. While Vanillic Acid possesses polar functional groups, its high hydrophilicity and relatively low polarizability (163.23 a.u.) make it more likely to remain solvated than to bind to the nanoparticle surface.

These results reveal a clear relationship between molecular structure, solubility, and surface binding. Compounds like Luteolin, Catechin, and Sinapic Acid, which exhibit a balanced hydrophilic-lipophilic profile, are more likely to form stable, high-affinity interactions with Er_2_O_3_ nanoparticles. This is supported by their lower HOMO–LUMO gaps, high electrophilicity, sharp RDF peaks, and stable RMSD values, all of which point to strong surface adsorption behavior. In contrast, highly hydrophilic (Vanillic Acid) or highly lipophilic but unreactive (Limonene, Sabinene) compounds demonstrate lower interaction energies and less favorable binding behavior.

## Conclusion

This study set out to investigate the molecular-level interactions between selected phytochemicals and Er_2_O_3_ nanoparticles using a combined Density Functional Theory (DFT) and Molecular Dynamics (MD) approach. By evaluating the electronic structure, reactivity descriptors, solubility profiles, and dynamic behavior of six representative compounds—Catechin, Luteolin, Sinapic Acid, Vanillic Acid, Limonene, and Sabinene—we aimed to identify phytochemicals with the highest potential for nanoparticle surface functionalization in biomedical applications.

The results successfully meet these objectives. Luteolin, Catechin, and Sinapic Acid emerged as the most promising candidates, exhibiting: i) High binding affinity with Er_2_O_3_ as demonstrated by strong interaction energies, ii) Stable adsorption behavior supported by low RMSD and sharp RDF peaks, iii) Favorable quantum descriptors, including low HOMO–LUMO energy gaps, high polarizability, and significant electrophilicity, and iv) Balanced hydrophilic-lipophilic profiles, ideal for nanoparticle conjugation in aqueous environments.

These compounds consistently showed stronger, more localized, and stable interactions with the Er_2_O_3_ surface, underpinned by their polar functional groups and moderate solubility, making them excellent candidates for applications such as drug delivery, biosensing, and photodynamic therapy.

In contrast, Vanillic Acid, while highly hydrophilic and polar, demonstrated weaker nanoparticle interactions due to its stronger affinity for water, limiting its surface-binding potential. Likewise, Limonene and Sabinene, despite being more lipophilic, showed limited interaction strength and electronic reactivity, restricting their suitability for nanoparticle-based applications.

Overall, this study confirms that the interplay between molecular polarity, solubility, and electronic reactivity governs the effectiveness of phytochemical-nanoparticle interactions. The findings not only provide a framework for rational selection of bioactive compounds for nanomaterial design but also contribute to the development of functional nanoplatforms tailored for enhanced therapeutic and diagnostic performance.

## Data Availability

No datasets were generated or analysed during the current study.
